# Delayed recovery of spontaneous circulation following cessation of cardiopulmonary resuscitation in an older patient: a case report

**DOI:** 10.1186/1752-1947-7-65

**Published:** 2013-03-12

**Authors:** Yili Huang, Sijun Kim, Amishi Dharia, Aleksander Shalshin, Jan Dauer

**Affiliations:** 1 Yale University School of Medicine, New Haven, CT, USA; 2 New York College of Osteopathic Medicine, Westbury, NY, USA; 3 New York-Presbysterian Hospital, New York, NY, USA; 4 North Shore-LIJ Plainview Hospital, Plainview, NY, USA

## Abstract

**Introduction:**

This report describes the apparent ‘resurrection’ of a patient in an emergency department setting. Befittingly named the ‘Lazarus phenomenon’, the recovery of spontaneous circulation after cessation of cardiopulmonary resuscitation is an extremely rare occurrence that was first described in 1982 and has been mentioned only 38 times in the medical literature. Our patient’s case is remarkable in that it helps illustrate many of the mechanisms of this rare phenomenon. It also serves as a reminder of our limitations in determining when to terminate cardiopulmonary resuscitation and suggests that cessation of cardiopulmonary resuscitation should be approached with more care.

**Case presentation:**

An 89-year-old Caucasian woman with a medical history of hypertension, atrial fibrillation, hypothyroidism, aortic insufficiency, lymphedema and hypoxia secondary to partial lung resection presented to our hospital after a witnessed fall unassociated with head trauma or loss of consciousness. On examination, our patient was saturating at 85 percent and exhibited a decreased range of motion of the upper extremities and left hip. Radiographic images revealed a left femoral neck and left distal radius fracture. Our patient was stabilized on 100 percent fraction of inspired oxygen and was awaiting transfer to an in-patient unit when, at 3:30 a.m., she went into cardiac arrest. An advanced cardiac life support protocol was initiated, at which time our patient was intubated and administered epinephrine, vasopressin and sodium bicarbonate. Our patient remained unresponsive and asystolic so cardiopulmonary resuscitation was abandoned at 3:48 a.m. After five minutes a ventricular contraction was noted at 3:51 a.m. This progressed to sinus rhythm with a pulse at 3:53 a.m. Our patient was stabilized on norepinephrine and moved to our Intensive Care Unit. At 10:55 a.m., however, our patient again arrested and, despite resuscitative efforts, was pronounced dead at 11:03 a.m.

**Conclusions:**

Our patient’s case clearly illustrates many of the proposed mechanisms for delayed return of spontaneous circulation including pulmonary hyper-inflation, hyperkalemia, delayed drug onset, and embolism dislodgement. Our patient represents a humbling and disturbing reminder that our medical acumen does not necessarily dictate the fate of our patients and that the decision to discontinue cardiopulmonary resuscitation should be approached with care by incorporating techniques such as end-tidal carbon dioxide, ventilator disconnect and passive monitoring.

## Introduction

Our patient’s case illustrates the recovery of spontaneous circulation (ROSC) after the cessation of cardiopulmonary resuscitation (CPR) attempts. This is a rare occurrence that was first described in 1982, and became known as the ‘Lazarus phenomenon’, appropriately named by Bray in 1993 after the biblical figure Lazarus, whom Jesus resurrected. Only a small number of these cases have been reported in the medical literature. Not only is this an exceedingly rare occurrence, but it may also be under-reported possibly due to a concern with medico-legal ramifications, fear of criticism, lack of physiological understanding of the mechanism, lack of complete documentation and psychological disbelief.

The exact mechanism of the Lazarus phenomenon is still unclear but there are various proposed mechanisms that may contribute to delayed ROSC: embolus or thrombus dislodgement, hyper-inflation, hyperkalemia, delayed action of drugs, and potentially the role of fat embolism syndrome (FES).

The current medical literature suggests that the overall incidence of FES in long bone fractures in particular is 2 percent to 5 percent [1]. FES is a rare condition in which lipid particles enter the circulatory system, occluding blood vessels. This leads to multi-system dysfunction and is associated with a high risk of morbidity and mortality. It was first described in 1873 in patients presenting with femoral fractures similar to that of our patient. Of the documented cases, 90 percent are associated with some form of trauma related to large bone fractures (femur and pelvis) and orthopedic surgery [2].

Clinically, FES follows an asymptomatic course followed by skin, pulmonary, and/or neurological manifestations. Symptoms follow 12 to 36 hours post-traumatic injury and primarily manifest as dyspnea, tachypnea and hypoxemia in up to 75 percent of cases [3]. The patient’s condition can progressively deteriorate, resulting in respiratory failure and severe acute respiratory distress syndrome [4]. It is important to note that hypoxemia may be detected hours before the onset of initial respiratory distress. A non-palpable petechial rash on the chest, axilla, neck, and conjunctiva is present in 60 percent of cases and manifests within 24 to 36 hours, lasting for up to seven days [5].

Levy and colleagues theorize that the initial presenting symptoms are explained by the occlusion of blood vessels with fat globules that are released from the marrow of traumatized or fractured long bones. As these particles occlude the pulmonary vasculature, fibrin and platelets adhere, while lipases continue to promote the release of free fatty acids. This sets off an inflammatory cascade and the subsequent endothelial damage progresses to acute respiratory distress, hemodynamic instability and cardiopulmonary arrest [6].

## Case presentation

An 89-year-old Caucasian woman, body mass index (BMI) 17.2, with a known medical history of hypertension, rate controlled atrial fibrillation, hypothyroidism, aortic insufficiency, lymphedema and hypoxia secondary to partial lung resection on home medications of warfarin, levothyroxine, metoprolol, montelukast, furosamide, ipratropium bromide, and fluticasone presented to North Shore-LIJ Plainview’s emergency department at 2:30 p.m. after a witnessed fall not associated with head trauma or loss of consciousness. Her vital signs on presentation were blood pressure of 144/81mmHg; pulse of 119 beats per minute; respiratory rate of 18 breaths per minute; temperature of 98 degrees Fahrenheit, and oxygen saturation of 85 percent on room air. Our patient’s baseline oxygen saturation was unknown and she denied being on home oxygen therapy. On physical examination our patient was not wheezing on presentation but revealed shortness of breath on room air with diffusely scattered rhonchi. An ipratropium-albuterol nebulizer was administered over 10 minutes, and our patient was then put on oxygen supplementation.

**Figure 1:**
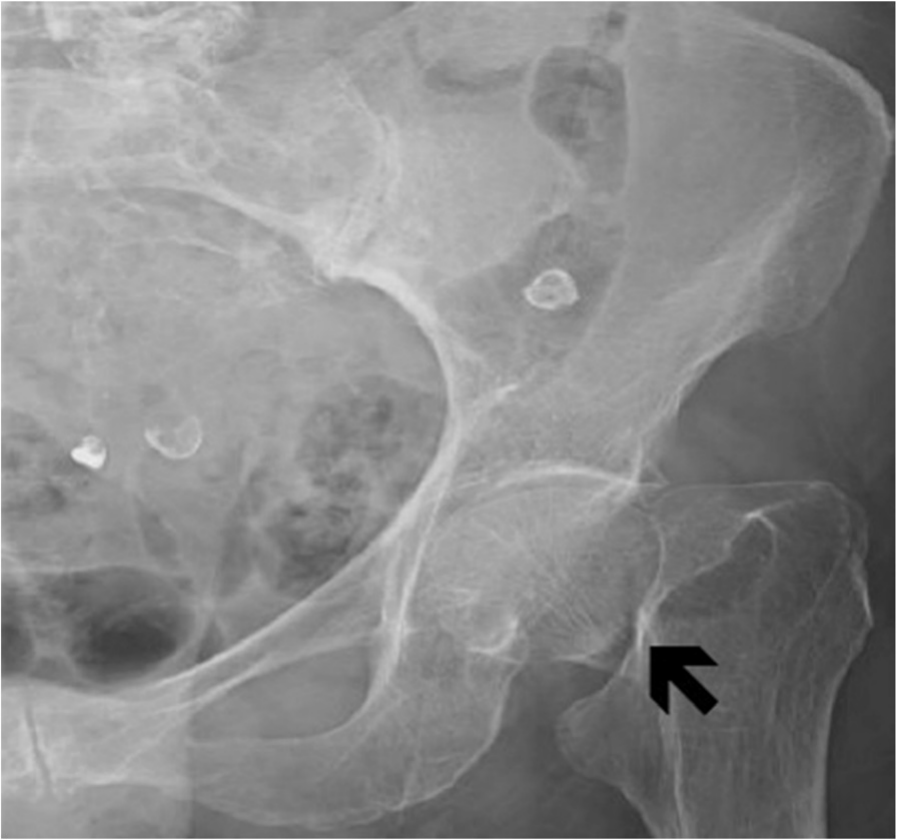
X-ray of the left hip and femur: a left subcapital femoral neck fracture (black arrow) can be seen.

**Figure 2:**
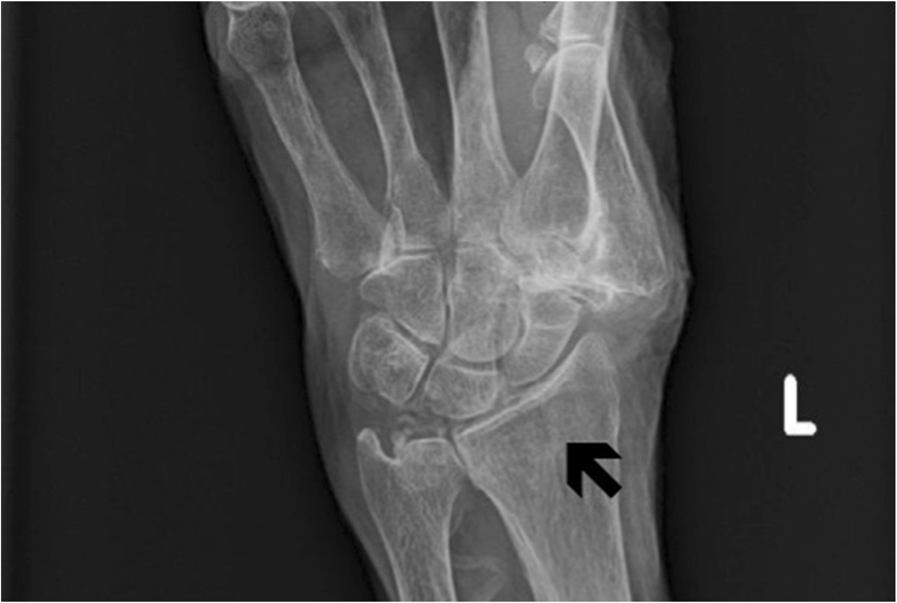
X-ray of the left wrist: a non-displaced fracture of the left distal radius (black arrow) can be seen.

On examination, our patient was alert and oriented to person, place and time but experienced a decreased range of motion of the upper extremities. The left lower extremity was shortened and externally rotated with at the hip joint. Arterial blood gas on room air showed a pH of 7.28 (reference range 7.38 to 7.42), partial carbon dioxide (CO_2_) (PCO_2_) of 44mmHg (reference range 35 to 45mmHg), calculated bicarbonate (HCO3) of 19mmHg (reference range of 22 to 29mmHg) partial oxygen (O_2_) (PO_2_) of 58mmHg (reference range of 80 to 100mmHg) and calculated base excess of −5.8 (reference range of −2.0 to 2.0). These results implied hypoxia with mixed metabolic and respiratory acidosis. Laboratory test results revealed a nearly therapeutic international normalized ratio (INR) of 1.93 with a basic metabolic profile including serum potassium within normal limits (4.1mEq/L). Our patient’s glucose level on arrival was 158mg/dL. An electrocardiogram (ECG) revealed atrial fibrillation at 113 beats/minute along with right bundle branch and left anterior fascicular block. We were unable to obtain a previous ECG for comparison. Her lactate level was not obtained at this time. Relevant imaging studies included a pelvic X-ray revealing a left subcapital femoral neck fracture (Figure 1), X-ray of the left wrist, which demonstrated a non-displaced fracture of the distal radius (Figure 2), and a portable chest X-ray showing partial right lung resection and bilateral haziness with questionable consolidation. Our patient’s respiratory status was stabilized on 100 percent fraction of inspired oxygen (FiO_2_), non-rebreather mask, and our patient was placed in a volar wrist splint to await transfer to a monitored in-patient bed.

At 3:30 a.m., our patient suddenly became bradycardic and progressed to asystole. She was found to be pale and pulseless. Cardiopulmonary resuscitation (CPR) was immediately begun following an Advanced Cardiac Life Support (ACLS) protocol. Initial respiratory support with a bag mask was attained and our patient’s trachea was secured with intubation at 3:32 a.m. As per the ACLS protocol, 1mg of intravenous epinephrine was given, followed by 40mg of intravenous vasopressin given at 3:36 a.m. Our patient received her second dose of 1mg epinephrine at 3:40 a.m. Finally at 3:46 a.m., our patient received one ampule of sodium bicarbonate. Our patient remained unresponsive to continued resuscitation efforts so CPR was abandoned at 3:48 a.m. Right femoral venous access had been attempted during the resuscitative effort, and cardiac monitors were unwittingly left on our patient during cleaning and removal. At 3:51, before the complete removal of the femoral venous catheter, a single ventricular contraction was noticed on the monitor. This single contraction progressed to a couplet, then a triplet and finally into a normal sinus rhythm with a palpable pulse at 3:53 a.m. Her initial blood pressure was measured at 80/50mmHg and our patient was started on a norepinephrine drip at 10μg/min through the femoral venous access. A right femoral arterial line was also established for continuous blood pressure monitoring. Her blood pressure stabilized at a mean arterial pressure of 70 and our patient was transferred to our Intensive Care Unit (ICU) at 4:30 a.m. In the ICU, a hypothermia protocol was initiated and vasopressors were titrated to maintain systolic blood pressure greater than 90mmHg. Unfortunately at 10:55 a.m. our patient went into ventricular tachycardia and, despite resuscitative efforts, our patient was pronounced dead at 11:03 a.m.

## Discussion

**Figure 3:**
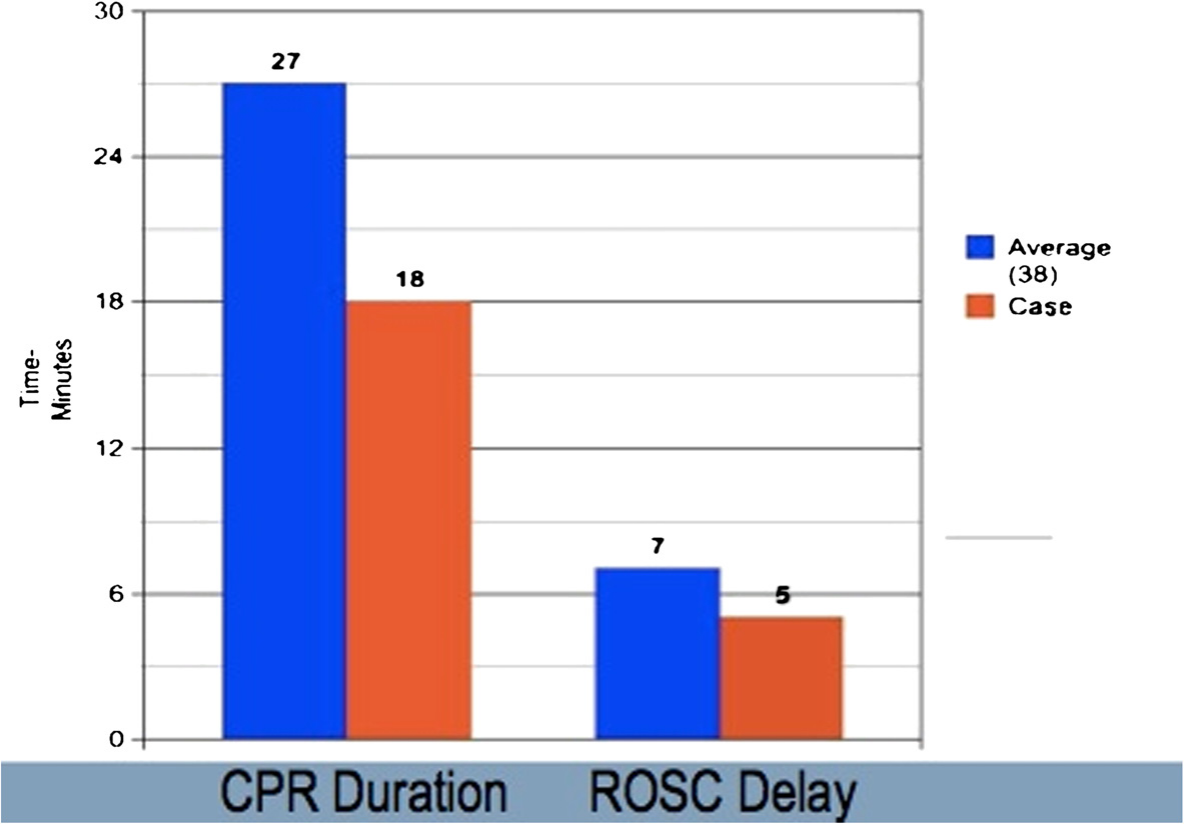
**Comparison of the average cardiopulmonary resuscitation duration (27 minutes) of the 38 documented cases with that of our patient’s case (18 minutes), as well as the average recovery of spontaneous circulation delay (7 minutes) of the documented cases with our patient’s case (5 minutes).** CPR, cardiopulmonary resuscitation; ROSC, recovery of spontaneous circulation.

**Figure 4:**
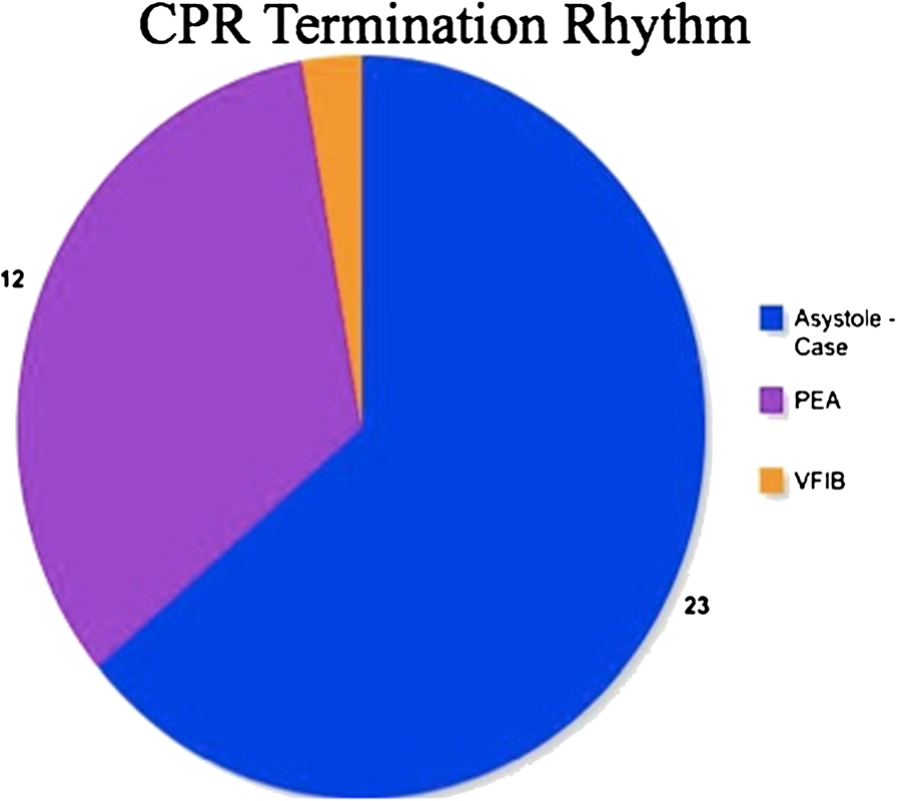
**Cardiopulmonary resuscitation termination rhythms.** CPR, cardiopulmonary resuscitation; PEA, pulseless electrical activity; VFIB, ventricular fibrillation.

With the exception of our patient’s case, only 38 cases of Lazarus phenomenon have been reported in the medical literature. The ages of these patients ranged from 27 to 94 years old [7] and the majority of the patients were diagnosed as having myocardial infarction or obstructive airway disease at the time of cardiopulmonary arrest. Average resuscitation efforts were about 27 minutes (Figure 3). CPR was terminated while 23 of the patients were asystolic, 12 with pulseless electrical activity (PEA) and one with ventricular fibrillation (Figure 4). In 82 percent of these patients, ROSC occurred within 10 minutes of cessation of CPR with an average ROSC delay of seven minutes (Figure 3). About half of those who achieved delayed ROSC were able to achieve good neurological recovery but the other half were unable to recover and died soon after. These outcomes did not correlate to duration of CPR, the time interval of delayed ROSC, or the pre-existing diagnosis [8].

Our patient is fairly typical in the context of the documented cases of delayed ROSC. Our patient was in the upper range for age but had good pre-morbid mental status and function. Although the cause of cardiopulmonary arrest is unclear, our patient did have a significant pre-existing cardiac and pulmonary dysfunction. The 18 minutes of CPR our patient received was less than the average 27 minutes (Figure 3), and our patient terminated in asystole (Figure 4). A total of five minutes elapsed between the termination of CPR and full ROSC (Figure 3). Unfortunately our patient was unable to recover and died seven hours later.

Given our patient’s history of chronic obstructive pulmonary disease, it can be argued that our patient’s hypoxic drive was removed as a result of being stabilized on 100 percent FiO_2_. However, our patient’s pulmonary status improved shortly after being treated for pain and as a result she was eventually weaned off oxygen support and our patient continued to maintain good cardiopulmonary status and adequate saturation while waiting for an in-patient bed.

Our patient received intravenous morphine in small increments of 2.5mg twice in a four-hour interval with good relief of pain and there was no evidence to suggest that our patient had developed respiratory depression as a consequence of these medications.

We postulate that our patient experienced FES as a consequence of long bone fractures that may have progressed to cardiopulmonary arrest. Proximal femur fractures as well as multiple fractures are associated with increased incidence of FES [9]. Although the exact cause of cardiopulmonary arrest is largely unclear, clinical evidence suggests that the fall and the subsequent fractures may have potentially precipitated FES. It is important to note, however, that a post-mortem study was not conducted and it is therefore difficult to ascertain that our patient’s arrest was a direct result of FES.

Evaluation of our patient revealed tachycardia (117 beats/minute) and hypoxia (oxygen saturation [SpO_2_]: 85 percent PaO_2_: 58mmHg on room air), which are consistent with the earliest manifestations of FES. Although these risk factors and the presentation may also suggest a far more common venous thromboembolic disease, our patient was on warfarin for the treatment of atrial fibrillation and her nearly therapeutic INR of 1.98 makes such a diagnosis unlikely. Given these risk factors and the clinical presentation, it is plausible that FES may have potentially played a role in our patient’s cardiopulmonary arrest.

Frölich described a case of spontaneous recovery of circulatory function after cessation of CPR in an intra-operative setting. A patient scheduled for endovascular stent graft prosthesis of an enlarging thoraco-abdominal aortic aneurysm suffered intra-operative cardiopulmonary arrest secondary to myocardial ischemia shortly after guidewire and catheter placement. Despite 43 minutes of CPR including defibrillation, chest compressions, epinephrine and sodium bicarbonate administration, resuscitative efforts were discontinued without ROSC. Unexpectedly, spontaneous circulation returned five minutes later. The authors postulated that the intra-operative myocardial ischemia was caused by acute obstruction of the left coronary artery, possibly by an endovascular plaque released by the guidewire manipulation within the thoracic aorta, and that this plaque was dislodged during CPR, which allowed for cardiac reperfusion [10]. Given our patient’s clinical presentation, it is feasible that resuscitative efforts may have dislodged a thromboembolus, which explains the delayed ROSC witnessed after the succession of CPR attempts.

During a resuscitation effort, the patient’s airway is usually supported by positive pressure ventilation either via a bag mask or mechanical ventilation. Dynamic hyper-inflation can occur when rapid positive pressure ventilation is given without adequate time for exhalation. This leads to an increase in intra-thoracic pressure that can then cause a decrease in venous return and therefore persistent circulatory failure. It is analogous to cardiac tamponade, in which the impediment to cardiac filling can lead to prolonged PEA. Only by reducing the intra-thoracic pressure can circulation be restored. Although it can theoretically occur in any patient receiving positive pressure ventilation with inadequate expiratory periods, this increase in auto-positive end expiratory pressure (PEEP) is most often seen in patients with obstructive pulmonary disease [11]. Lapinsky and Leung showed that a majority of patients without clear explanations for PEA (13 of 18) had a history of obstructive pulmonary pathology, and a significant number (3 of 13) experienced delayed ROSC [12].

The effects of auto-PEEP could have also very well been a plausible explanation in our patient’s case. Our patient’s exact social and medical history are unclear, however, our patient presumably did suffer from obstructive pulmonary disease based on the fact that our patient was managed with common chronic obstructive pulmonary disease medications, such as inhaled budesonide and ipratropium. As per the ACLS protocol, positive pressure ventilation was provided with the initiation of cardiopulmonary resuscitation, first via bag-mask and subsequently via endotracheal tube. Our patient’s auto-PEEP was not measured while our patient was on the ventilator and therefore could have potentially built up during CPR because our patient was being mask ventilated. It is plausible that these positive pressure breaths may have stacked to the point where pre-load and cardiac output was impeded. Adequate exhalation would have been impossible until respiratory support terminated when resuscitation efforts were abandoned. The five minutes of delayed ROSC could have been the time necessary for the thoracic pressures to equilibrate to the point where sufficient cardiac output and spontaneous circulation was once again possible. During those five minutes, our patient could have had subclinical ROSC (where there is a minute level of spontaneous circulation that is unrecognized by our monitors). Furthermore, having been ventilated with 100 percent oxygen during the resuscitation effort, there would have been enough oxygen reserves in the residual volume of the lungs to support her oxygen demands during this period of unrecognized ROSC.

Another proposed mechanism for delayed ROSC is hyperkalemia. Potassium is the most abundant intracellular cation and shifts in its concentration may lead to prolonged cardiopulmonary arrest, and leave the myocardium refractile for some time [13]. The most striking example of this was documented by Kao and colleagues, describing a 68-year-old patient who developed cardiac arrest secondary to hyperkalemia and did not respond to over 100 minutes of CPR, but instead responded to hemodialysis and progressed to a full neurologic recovery [14]. Although the effects of sodium bicarbonate on potassium are transient, it may play a contributing role in the delayed ROSC by driving potassium intracellularly. Voelckel and Kroesen hypothesized this as a mechanism of delayed ROSC [15].

Although our patient’s serum potassium was within normal limits at 4.1mEq/L (normal 3.5 to 4.5mEq/L), treatment with metoprolol and digoxin did put her at risk for hyperkalemia. Of note, our patient was also on furosemide and given sodium bicarbonate 10 minutes into the resuscitative efforts which may have driven potassium intracellularly. Given our patient’s presentation of atrial fibrillation on her admission ECG and normal serum potassium level, digoxin toxicity does not appear to be contributory in our patient’s morbidity and mortality.

Delayed action of the resuscitation drugs given could be another explanation for the delayed ROSC. Drugs were administered via a peripheral intravenous line. Decreased venous return caused by dynamic hyper-inflation may have affected central delivery of the drugs resulting in a lag time in its resuscitative effects.

A post-mortem study was not conducted and the actual cause of the cardiovascular arrest remains to be debated.

## Conclusions

Lazarus syndrome is a rare phenomenon that can be perplexing to the health care provider and brings to question the appropriate timing of the decision to discontinue CPR. It is humbling to know that our medical judgment does not necessarily dictate the fate of a patient in cardiac arrest, and that so much of our understanding is still theoretical. It is also a disturbing reminder that in these cases, so many factors outside our control can lead to serious professional and legal consequences.

Although our patient did not make a meaningful neurologic recovery and died eight hours later, about 45 percent (17 of 38 patients) of documented cases of delayed ROSC did achieve good neurological recovery and 35 percent (14 of 38) were eventually discharged home without any significant neurological sequelae [5]. Our patient’s case illustrates that cessation of CPR should be approached with care. One of the tools that may be helpful is capnography. The use of end tidal carbon dioxide measurement during CPR has been included in the most recent revision of the ACLS guidelines, but is still not widely available and practiced. Values above 10 to 15mmHg indicate a favorable prognosis and should preclude cessation of CPR. Also, given that one of the most accepted mechanisms of delayed ROSC is dynamic hyper-inflation, we recommend disconnecting the ventilator as a last ditch effort in patients who are unresponsive to resuscitative efforts [3].

Our patient’s case also demonstrates the need to further examine the potential implications of FES in its relation to the current standards of patient resuscitation. Finally, because delayed ROSC occurred within 10 minutes of cessation of CPR, in most cases when monitors are left connected we recommend passive monitoring for a minimum of 10 minutes following the cessation of CPR.

## Consent

Written informed consent was obtained from the patient’s next of kin for publication of this case report and any accompanying images. A copy of the written consent is available for review by the Editor-in-Chief of this journal.

## Abbreviations

ACLS: Advanced cardiac life support; CPR: Cardiopulmonary resuscitation; ECG: Electrocardiogram; FES: Fat embolism syndrome; ICU: Intensive care unit; INR: International normalized ratio; PEA: Pulseless electrical activity; PEEP: Positive end expiratory pressure; ROSC: Return of spontaneous circulation.

## Competing interests

The authors declare that they have no competing interests.

## Authors’ contributions

YH was the primary author and designed the study, analyzed the data, and prepared the manuscript. SK helped analyze the data, described the case study and edited copies of the manuscript. AD helped analyze the data and described the case study. AS helped edit the manuscript. JD is the primary attending and helped edit the manuscript. All authors have read and approved the final version of this manuscript.
